# Syphilis and HIV prevalence and associated factors to their co-infection, hepatitis B and hepatitis C viruses prevalence among female sex workers in Rwanda

**DOI:** 10.1186/s12879-017-2625-0

**Published:** 2017-07-28

**Authors:** Mwumvaneza Mutagoma, Laetitia Nyirazinyoye, Dieudonné Sebuhoro, David J. Riedel, Joseph Ntaganira

**Affiliations:** 1 Rwanda Biomedical Centre, Ministry of Health, P. O. Box, 7162 Kigali, Rwanda; 2 University of Rwanda, College of Medicine and Health Sciences, School of Public Health, Kigali, Rwanda; 3 Institute of Human Virology and Division of Infectious Diseases, University of Maryland School of Medicine, Baltimore, MD USA

**Keywords:** Female sex workers, HIV, Syphilis, HBV, HCV, Co-infection, Rwanda

## Abstract

**Background:**

Human Immunodeficiency Virus (HIV), syphilis, Hepatitis B Virus (HBV) and Hepatitis C Virus (HCV) are sexually transmitted infections (STIs) and share modes of transmission. These infections are generally more prevalent among female sex workers (FSWs).

**Methods:**

This is a cross-sectional study conducted among female sex workers (FSWs) in Rwanda in 2015. Venue-Day-Time (VDT) sampling method was used in recruiting participants. HIV, syphilis, HBV, and HCV testing were performed. Descriptive analyses and logistic regression models were computed.

**Results:**

In total, 1978 FSWs were recruited. The majority (58.5%) was aged between 20 and 29 years old. Up to 63.9% of FSWs were single, 62.3% attained primary school, and 68.0% had no additional occupation beside sex work. Almost all FSWs (81.2%) had children. The majority of FSWs (68.4%) were venue-based, and most (53.5%) had spent less than five years in sex work. The overall prevalence of syphilis was 51.1%; it was 2.5% for HBV, 1.4% for HCV, 42.9% for HIV and 27.4% for syphilis/HIV co-infection. The prevalence of syphilis, HIV, and syphilis + HIV co-infection was increasing with age and decreasing with the level of education. A positive association with syphilis/HIV co-infection was found in: 25 years and older (aOR = 1.82 [95% CI:1.33–2.50]), having had a genital sore in the last 12 months (aOR = 1.34 [95% CI:1.05–1.71]), and having HBsAg-positive test (aOR = 2.09 [1.08–4.08]).

**Conclusion:**

The prevalence of HIV and syphilis infections and HIV/syphilis co-infection are very high among FSWs in Rwanda. A strong, specific prevention program for FSWs and to avert HIV infection and other STIs transmission to their clients is needed.

## Background

Human Immunodeficiency Virus (HIV), syphilis, Hepatitis B Virus (HBV) and Hepatitis C Virus (HCV) are sexually transmitted diseases (STIs) and have shared modes of transmission. Female sex workers (FSWs) are at high risk to contract STIs due to the work and their behavior [1, 2]. The World Health Organization (WHO) reported that the global prevalence of HIV among FSWs was 11.8% with significant variation by region, with the highest prevalence found in sub-Saharan Africa with an aggregated prevalence of 36.9% [3]. The prevalence of HIV among FSWs is 10–20-fold higher than the general population in many African countries [4].

A meta-analysis study conducted in African countries found that the HIV prevalence among FSWs varied between 19% and 60% [5]. Despite the control of HIV among the general population in Rwanda (3.0%) [6], STIs are still a concern among FSWs. In 2011 a 24.0% prevalence of HIV was estimated among FSWs in Kigali, Rwanda [7] which was three times higher than the prevalence of HIV in the general population in the same city (7.3%) [8] in the same period.

WHO reported that 10 countries had greater than 10% syphilis prevalence among sex workers, while some countries reported a syphilis prevalence of more than 20% [1]. A low prevalence of syphilis among FSWs was reported in few African countries. In Somaliland, the prevalence of syphilis was 3.1% [9], and 3.3% among FSWs in Kisumu, Kenya [10]. A high prevalence of syphilis among FSWs was reported in other countries – for example, it was 21.0% in Kampala, Uganda [11] and 52.4% in Addis Ababa, Ethiopia [12] in two separate cross-sectional studies.

Few data about HCV and HBV among FSWs have been published. WHO estimated the worldwide number of people living with chronic HCV between 130 and 150 million [13] while this number was estimated at 240 million of people chronically infected with hepatitis B [13]. In China, the prevalence of HCV was estimated at 0.8% in one province [14] and 1.1% in another province [15], it was 2.8% in India [16].

The prevalence of HBV in India was estimated at 7.6% [17] while it was 17.1% in Nigeria [17].

The interaction between syphilis and HIV is not well documented [18]. Syphilis as an ulcerative infection, increases HIV transmission. Syphilis in clinically immunosuppressed HIV-infected patients is reported to be associated with greater organ involvement [19]. In Nepal the proportion of syphilis and HIV co-infection was 31.0% [20]. Factors such as STIs symptoms, lower level of education were described being positively associated with syphilis and HIV infections [21].

Currently, few data are published on HBV and HCV among FSWs in Rwanda. A study conducted in 2013 found that the prevalence of HBV was 1.6% among blood donors, while the prevalence of HCV was 2.9% among health workers [22]. A meta-analysis of studies from sub-Saharan Africa reported an HBV prevalence of 4.1% and an HCV prevalence of 2.1% among HIV-infected persons [23].

The aim of this study is to describe the burden of syphilis, HIV, HBV, HCV and HIV/syphilis co-infection and associated factors among FSWs in Rwanda.

## Methods

### Study setting and population

This study was a cross-sectional behavioral and biological survey among FSWs in Rwanda in 2015. Venue-Day-Time (VDT) sampling method was used to identify specific places and times where FSWs await their subsequent sexual clients [24]. The time frame was determined by the peak hours of the presence of FSWs at hot spots.

Before the data collection process, a rapid assessment to estimate the number of expected FSWs was conducted. The number of FSWs at each site was estimated by key informants selected in the same target population. The survey was conducted in all hot spots with 5 or more FSWs per day. The number of participants recruited in each hot spot was determined using probability proportional to size of each selected site.

### Population and inclusion criteria

Self-reported FSW aged 15 years or more found in a FSW hot spot area identified by a FSW key informant was eligible to participate. Excluded from the survey were: FSWs less than 15 years old, not self-identifying as a female sex worker, declining to consent to participate in the survey.

### Sampling

A two-stage sampling method using VDT sampling, referring to the specific peak time and place that FSWs were present at their hot spot was used. The primary sampling unit was the time and place where FSWs were present at the hotspot, and the secondary sampling unit was FSWs in the selected time and place. A “take-all” approach sampling was used. Once the expected sample size at the site was obtained, data collection was closed at that site. For primary sampling unit (PSU) selection, 180 hotspots were selected from the sampling frame in the country.

### Sample size calculation

The sample size calculation used the Z score value of 1.96 with an alpha level of 0.05 (95% confidence), the design effect of 3.2, a relative 10% precision around a point estimate (HIV prevalence) of 51% among female sex workers in 2010. A minimum sample size of 1980 FSWs was calculated.

### Data collection methods

FSWs were contacted by data collectors to take part in the survey. If eligible, a verbal informed consent was administered with no identifying information. After verbal consent was received, pre-test counselling was conducted by a nurse counsellor. The laboratory technician drew blood by venipuncture. A Personal Digital Assistant (PDA) questionnaire was administered by survey staff. Rapid diagnostic test results were returned to the participant and post-test counselling was conducted in few minutes. Participants whose test was positive for HIV, syphilis, HBV or HCV were referred to the health centre of the patient’s choice for clinical care. Blood samples were transported to the National Reference Laboratory (NRL) for confirmatory HIV testing for survey purposes.

### HIV testing

HIV rapid testing was performed by a trained laboratory technician using the nationally approved algorithm of three serial rapid tests (currently approved protocol: Shanghai-Kehua, Determine, and Uni-Gold). For the survey purpose, ELISA-based testing was used (Vironostika® HIV Uni-form II Ag/Ab, 4th Generation) for all samples. For samples testing positive with ELISA, a confirmatory test used a second EIA called Murex HIV Ag/Ab Combination 4th Generation Murex®.

### Syphilis testing

Screening for syphilis was conducted on site using the SD BIOLINE SYPHILIS 3.0 rapid test to detect syphilis anti-bodies. A reacting sample was retested using the RPR test at NRL. A sample that was positive for both tests was recorded as positive; a non-reacting sample to the second test was recorded as negative.

### Hepatitis B testing

Screening for HBV was performed using the SD BIOLINE HBV rapid test to detect HBV surface antigen (HBsAg). A reacting sample was recorded as positive.

### Hepatitis C testing

Screening for HCV was performed using the SD BIOLINE HCV rapid test. The test detected HCV antibody (anti-HCV). A reacting sample was recorded as positive.

All FSWs with positive tests for any test were transferred to the nearest health facility or the health facility of their choice in order to provide quick results for clinical care.

### Data analysis

STATA 13 software was used for data analysis. Mean, proportion, and 95% confidence interval were computed for descriptive analysis of variables. Prevalence in different socio-demographic characteristics was estimated for HIV, syphilis, HBV, HCV infections and for HIV and syphilis co-infection.

HIV and syphilis co-infection associated factors significant at the *p* < 0.05 level in bivariate analysis were included in a multivariable logistic regression model. Multivariable analysis was computed to determine the effect of independent variables to HIV and syphilis co-infection. Variables were retained in the final model when achieving *p* < 0.05 significance.

## Results

Table 1 displays the description of respondents in the survey. In total, 1978 FSWs participated in the survey. The median age was 26 years old (Interquartile Range [IQR] 22,31). The majority (58.5%) was aged between 20 and 29 years old. Up to 63.9% of FSWs were single and 30.0% were either divorced or separated. A majority (68.4%) of FSWs were recruited in and around venues (e.g. bars, night clubs, and hotels). About formal education, 62.3% attained primary school. Most FSWs (68.0%) had no other occupation beside sex work. The majority (56.4%) of FSWs had health insurance. Almost all FSWs (81.2%) had children; the average number of children per participant was two. The average monthly income was Rwf 40,549.0 ($50.20).

**Table 1:** Background characteristics among sex workers, 2015 Rwanda

Characteristics	Number	Percent
Total	1978	100
Median age	26	
Age group 1		
15–19	198	10.0
20–24	618	31.3
25–29	538	27.2
30–34	360	18.2
35–39	159	8.1
40 +	102	5.2
Age group 2		
15–24	816	41.3
25 +	1159	58.7
Current marital status		
Single	1263	63.9
Married/cohabitating	19	1.0
Divorced/Separated	594	30.0
Widow	102	5.2
Province		
East	646	32.7
Kigali city	293	14.8
North	389	19.7
West	324	16.4
South	326	16.5
Hotspot type		
Road/public place	625	31.6
Venue-based	1353	68.4
Level of education		
None	353	17.9
Primary	1231	62.3
Secondary/Higher	394	19.9
Religion		
Catholic	775	39.2
Protestant	584	29.5
Muslin	164	8.3
Adventist	186	9.4
Traditional/Other/No religion	269	13.6
Occupation		
Sex work only	1345	68.0
Sex work with additional occupation	633	32.0
Additional occupation		
Small business at the market	94	4.8
Job in a bar	51	2.6
Street vendor	113	5.7
Job in a restaurant	10	0.5
House worker	22	1.1
Handicraft maker	23	1.2
Dressmaker	13	0.7
Hairdresser	41	2.1
Student	3	0.2
Other occupations	299	15.1
Had a valid health insurance		
Yes	1116	56.4
Had children		
Yes	1607	81.2
Duration of sex work		
0–5	1036	53.5
6–15	741	38.3
16+	159	8.2

Table 2 displays the prevalence of RPR, HBsAg, HCV Ab, HIV positive tests and syphilis and HIV co-infection. The overall prevalence of RPR-positive test among FSWs was 51.1%. This prevalence was increasing with age up to 29 years old. It was 42.9% [95% CI:36.0–49.9] in 15–19 years age group and 55.5% [95% CI:51.3–59.7] among 25–29 years age group. There was no significant difference in prevalence of RPR-positive test by marital status. The western (58.6% [95% CI:53.3–64.0]) and southern provinces (58.8% [95% CI:50.4–61.2]) had higher RPR-positive prevalence than the North(44.2% [39.3–49.2]). The prevalence of RPR-positive test was significantly decreasing with the level of education: it was 66.3% [95% CI:61.3–71.2], 51.9% [95% CI:49.1–54.7] and 34.9% [95% CI:30.1–39.6], respectively, in none, primary and secondary/higher education levels. RPR-positivity was increased with the duration of sex work: 47.8% [95% CI:44.7–50.8] and 54.9% [95% CI:51.3–58.5], respectively, among FSW with 0–5 years and 6–15 years of duration of sex work.

**Table 2:** Syphilis, HBV, HCV and HIV prevalence and HIV co-infection among FSW in Rwanda, 2015

Characteristics	Syphilis		HBV		HCV		HIV		Syphilis and HIV co-infection	
	*n*	% [95% CI]	*n*	% [95% CI]	*n*	% [95% CI]	*n*	% [95% CI]	*n*	% [95% CI]
Overall	1010	51.1 [48.9–53.3]	50	2.5 [1.8–3.2]	28	1.4 [0.8–1.9]	819	42.9 [39.5–43.8]	528	27.4 [25.4–29.4]
Age group										
15–19	85	42.9 [36.0–49.9]	6	3.0 [0.6–5.4]	3	1.5 [0.2–3.2]	49	22.3 [16.5–28.2]	25	13.0 [8.2–17.7]
20–24	292	47.3 [43.3–51.2]	12	1.9 [0.8–3.0]	6	1.0 [0.2–1.7]	213	32.7 [29.0–36.4]	129	21.3 [18.0–24.6]
25–29	298	55.5 [51.3–59.7]	12	2.2 [1.0–3.5]	3	0.6 [0.1–1.2]	243	43.2 [38.9–47.4]	148	28.5 [24.6–32.4]
30–34	198	55.0 [49.8–60.2]	14	3.9 [1.9–5.9]	5	1.4 [0.2–2.6]	195	55.0 [49.8–60.2]	137	39.3 [34.1–44.4]
35–39	86	54.1 [46.3–61.9]	4	2.5 [0.1–5.0]	8	5.0 [1.6–8.5]	85	53.8 [45.9–61.7]	58	36.9 [29.3–44.6]
40+	49	48.0 [38.2–57.9]	2	2. 0[0.8–4.7]	2	2.0 [0.8–4.7]	63	60.4 [50.7–70.1]	31	31.3 [22.0–40.6]
Age group 2										
15–24	377	46.2 [42.8–49.6]	18	2.2 [1.2–3.2]	9	1.1 [0.4–1.8]	262	30.2 [27.0–33.3]	154	19.3 [16.6–22.0]
25 +	631	54.5 [51.6–57.4]	32	2.8 [1.8–3.7]	18	1.6 [0.8–2.3]	586	49.8 [46.9–52.7]	374	33.3 [30.5–36.0]
Current marital status										
Single	629	49.8 [47.0–52.6]	28	2.2 [1.4–3.0]	17	1.4 [0.7–2.0]	495	37.5 [34.9–40.2]	314	25.4 [23.0–27.8]
Married/cohabitating	12	63.2 [39.3–87.0]	1	5.3 [0.1–16.3]	1	5.3 [0.0–16.3]	9	42.1 [17.7–66.6]	7	38.9 [13.9–63.8]
Divorced/Separated	319	53.7 [49.7–57.7]	17	2.9 [1.5–4.2]	8	1.4 [0.4–2.3]	285	47.3 [43.2–51.3]	172	30.0 [26.3–33.8]
Widow	50	49.5 [39.6–59.4]	4	3.9 [0.1–7.8]	2	2.0 [0.0–4.7]	59	59.4 [49.7–69.1]	35	35.7 [26.1–45.4]
Province										
East	320	49.6 [45.7–53.5]	17	2.6 [1.4–3.9]	12	1.9 [0.8–2.9]	243	36.3 [32.6–40.0]	136	21.6 [18.4–24.8]
Kigali city	146	49.8 [44.1–55.6]	8	2.7 [0.9–4.6]	2	0.7 [0.3–1.6]	157	54.3 [48.5–60.0]	102	35.7 [30.1–41.2]
North	172	44.2 [39.3–49.2]	7	1.8 [0.5–3.1]	6	1.5 [0.3–2.8]	143	34.8 [30.0–39.6]	84	21.7 [17.6–25.8]
West	190	58.6 [53.3–64.0]	10	3.1 [1.2–5.0]	5	1.5 [0.2–2.9]	147	44.4 [39.0–49.9]	102	32.6 [27.4–37.8]
South	182	55.8 [50.4–61.2]	8	2.5 [0.8–4.2]	3	0.9 [0.1–2.0]	158	46.3 [40.8–51.7]	104	33.6 [28.3–38.8]
Hotspot type										
Street-based	327	52.3 [48.4–56.2]	11	1.8 [0.7–2.8]	4	0.6 [0.0–1.3]	267	41.2 [37.3–45.1]	166	27.5 [23.9–31.1]
Venue-based	683	50.5 [47.8–53.2]	39	2.9 [2.0–3.8]	24	1.8 [1.1–2.5]	581	41.8 [39.2–44.5]	362	27.4 [25.0–29.8]
Level of education										
None	234	66.3 [61.3–71.2]	9	2.6 [0.9–4.2]	7	2.0 [0.5–3.4]	184	52.6 [47.3–57.8]	135	35.7 [34.5–44.9]
Primary	639	51.9 [49.1–54.7]	32	2.6 [1.7–3.5]	16	1.3 [0.7–1.9]	517	42.1 [39.3–44.9]	333	27.7 [25.1–30.2]
Secondary/Higher	137	34.9 [30.1–39.6]	9	2.3 [0.8–3.8]	5	1.3 [0.2–2.4]	118	30.3 [25.7–34.9]	60	15.7 [12.0–19.4]
Religion										
Catholic	388	50.1 [46.5–53.6]	19	2.5 [1.4–3.5]	13	1.7 [0.8–2.6]	327	41.8 [38.3–45.3]	207	27.3 [24.1–30.5]
Protestant	311	53.3 [49.3–57.4]	13	2.2 [1.0–3.4]	10	1.7 [0.7–2.8]	249	40.1 [36.1–44.1]	152	26.7 [23.1–30.4]
Muslin	80	48.8 [41.0–56.5]	3	1.8 [0.2–3.9]	1	0.6 [0.0–1.8]	67	42.1 [34.4–49.7]	50	31.7 [24.3–39.0]
Adventist	93	50.0 [42.7–57.3]	6	3.2 [0.7–5.8]	1	0.5 [0.0–1.6]	86	42.7 [35.5–50.0]	43	24.6 [18.1–31.0]
Traditional/Other/No religion	138	51.3 [45.3–57.3]	9	3.4 [1.2–5.5]	3	1.1 [0.0–2.4]	119	43.7 [37.7–49.6]	76	28.7 [23.2–34.2]
Had children										
Yes	826	51.4 [49.0–53.9]	44	2.7 [1.9–3.5]	23	1.4 [0.8–2.0]	718	43.7 [41.3–46.2]	444	28.4 [26.2–30.7]
No	184	49.6 [44.5–54.7]	6	1.6 [0.3–2.9]	5	1.4 [0.2–2.5]	130	32.6 [27.8–37.4]	84	23.1 [18.8–27.5]
Occupation										
Sex work only	701	52.1 [49.4–54.8]	37	2.8 [1.9–3.6]	24	1.8 [1.1–2.5]	591	42.9 [40.2–45.5]	366	28.9 [25.5–30.3]
Sex work with additional occupation	309	48.9 [45.0–52.8]	13	2.1 [0.9–3.2]	4	0.6 [0.0–1.3]	257	39.0 [35.2–42.8]	162	26.4 [22.9–29.9]
Duration of sex work										
0–5	495	47.8 [44.7–50.8]	22	2.1 [1.2–3.0]	12	1.2 [0.5–1.8]	347	33.7 [30.8–36.6]	220	21.7 [19.2–24.3]
6–15	406	54.9 [51.3–58.5]	25	3.4 [2.1–4.7]	10	1.4 [0.5–2.2]	352	47.8 [44.1–51.4]	233	32.6 [29.2–36.1]
16+	89	56.0 [48.2–63.8]	3	1.9 [0.3–4.0]	6	3.8 [0.8–6.8]	97	61.0 [53.3–68.7]	60	38.2 [30.5–45.9]

The prevalence of HBsAg-positive test among FSWs was 2.5% [95% CI:1.8–3.2]; the prevalence of HCAb-positive test among FSWs was 1.4% [95% CI:0.8–1.9].

The overall prevalence of HIV was 42.9% [95% CI:39.5–43.8]. This prevalence was increasing with age up to 34 years old. It was 22.3% [95% CI:16.5–28.2] among FSWs 15–19 years age group while it was more than two times higher among FSWs with 30–34 years old. The HIV prevalence was higher among divorced or separated (47.3% [95% CI:43.2–51.3]) and widowed (59.4% [95% CI:49.7–69.1]) compared to single (37.5% [95% CI:34.9–40.2]) FSWs. By province, the highest prevalence of HIV was in the city of Kigali (54.3% [95% CI:48.5–60.0]), the lowest was found in the Eastern province (36.3% [95% CI:32.6–40.0]). The prevalence of HIV was significantly decreasing with the level of education. It was 52.6% [95% CI:34.5–44.9], 42.1% [95% CI:39.3–44.9] and 30.3% [95% CI:25.7–34.9] respectively in none, primary and secondary/higher education levels. The prevalence of HIV was significantly increasing with the duration of sex work. It was 33.7% [95% CI:30.8–36.6], 47.8% [95% CI:44.1–51.4] and 61.0% [95% CI:53.3–68.7] respectively among FSW with 0–5 years, 6–15 and more than 16 years of duration of sex work (Table 2).

The prevalence of syphilis/HIV co-infection among FSWs was 27.4% [95% CI:25.4–29.4] while the proportion of RPR-positive test among HIV-positive was 69.2% [95% CI:66.1–72.4] and the proportion of HIV-positive among RPR-positive test was 56.4% [95% CI:53.3–59.5]. The prevalence of syphilis/HIV co-infection among FSWs increased with age up to 34 years old. It was 13.0% [95% CI:8.2–17.7] among FSWs 15–19 years age group and it was more than three times higher among FSWs 30–34 years 39.3% [95% CI:34.1–44.4]. The city of Kigali had the highest prevalence of syphilis and HIV co-infection (35.7% [95% CI:30.1–41.2]) compared to the Eastern and the Northern provinces with respectively (21.6% [95% CI:18.4–24.8]) and 21.7% [95% CI:17.6–25.8]. By level of education the prevalence of syphilis and HIV co-infection decreased with the level of education. It was 37.5% [95% CI:34.5–44.9], 27.7% [95% CI:25.1–30.2] and 15.7% [95% CI:12.0–19.4] respectively in none, primary and secondary/higher education levels. The prevalence of syphilis and HIV co-infection increased with the duration of sex work. It was 21.7% [95% CI:19.2–24.3] and 32.6% [95% CI:29.2–36.1] respectively among FSWs with 0–5 years and 6–15 years duration of sex work (Table 2).

In bivariable logistic regression, age 25 years or older, being ever married, having more than five years of duration of sex work, having children, having had genital sores in the last 12 months, and having HBsAg positive test were positively associated with syphilis and HIV co-infection, whereas secondary or high education level was negatively associated with syphilis and HIV co-infection (Table 3).

**Table 3:** Factors associated with HIV and syphilis co-infection among FSWs in Rwanda, 2015

Characteristics	Bivariable			Multivariable		
	OR	95% CI	*p*-value	aOR^a^	95% CI	*p*-value
Age group						
15–24 years	1.00			1.00		
≥ 25 years	2.09	1.68–2.56	**0.00**	1.82	1.33–2.50	**0.00**
Marital status						
Never married	1.00			1.00		
Ever married	1.32	1.08–1.63	**0.01**	1.01	0.78–1.32	0.09
Education						
None/primary	1.00			1.00		
Secondary and higher	0.50	0.36–0.66	**0.00**	0.50	0.36–0.69	**0.00**
Occupation						
Sex work with additional occupation	1.00					
Sex work only	1.08	0.87–1.34	0.50			
Duration of sex work						
< 5 years	1.00			1.00		
≥ 5 years	1.82	1.49–2.40	**0.00**	1.22	0.92–1.61	0.39
Having children						
Doesn’t	1.00			1.00		
Had children	1.32	1.01–1.72	**0.04**	1.03	0.73–1.46	0.85
Recruitment based						
Venue based	1.00					
Street based	1.00	0.81–1.25	0.97			
Consistent condom use in the last 30 days						
Consistent	1.00					
Not consistent	1.00	0.82–1.33	1.00			
Drunk alcohol every day in the last 4 weeks						
Drunk alcohol	1.00					
Didn’t drink alcohol	0.88	0.71–1.08	0.23			
HIV Comprehensive knowledge^b^						
Had not	1.00					
Had	1.06	0.86–1.31	0.58			
Had genital sore in last 12 months						
Had not	1.00			1.00		
Had	1.32	1.08–1.63	**0.01**	1.34	1.05–1.71	**0.02**
Anal sex						
Had not	1.00					
Had	1.00	0.69–1.44	0.99			
HBV positive test						
Negative	1.00			1.00		
Positive	2.13	1.21–3.75	**0.01**	2.09	1.08–4.06	**0.03**
HCV positive test						
Negative	1.00					
Positive	1.26	0.57–2.80	0.57			

^a^OR = Adjusted odds ratio

^b^HIV Comprehensive knowledge was defined as knowing HIV prevention methods and rejecting misconception of HIV transmission

Only significant values (*p*-value) are in bold

In multiple logistic regression, FSWs with the following characteristics were positively associated with syphilis/HIV co-infection: age 25 years and older (aOR = 1.82 [95% CI:1.33–2.50]) compared to those less than 25 years old, having had a genital sore in the last 12 months (aOR = 1.34 [95% CI:1.05–1.71]) compared to FSWs without STI symptoms, and having HBsAg-positive test (aOR = 2.09 [1.08–4.08]) compared to FSWs with HBsAg-negative test (Table 3).

## Discussion

FSWs are among key drivers of HIV and other STIs in many countries. The current study was conducted among self-identified FSWs at different hot spots in the country to characterize the scope of this public health problem in Rwanda.

Two important findings resulted from this survey. Syphilis and HIV prevalence among FSWs in Rwanda was very high and increased with age and the duration of sex work. FSWs with any positive test were transferred to the nearest health facility or the health facility of choice for appropriate clinical care.

Overall HBV and HCV prevalence among FSWs in Rwanda was low (<5% each). Older FSWs, secondary or higher education, STIs symptoms, and HBV infection are independent predictors of HIV and syphilis co-infection.

Compared to the prevalence of the general population (3.0%) [6], the HIV prevalence among FSWs (42.9%) of our study was more than 14 times higher; and it is among the highest reported to date in Africa [3]. In the Rwandan context, sex work is illegal and often considered a shameful activity, some among FSWs operate in hidden unsecured places due to poor socio-economic conditions. This high HIV prevalence may be attributed to inconsistency in condom use, lack of negotiating power with clients over condom use, sexual violence and access to HIV care and treatment. In the current study, a proportion of 81.2% among FSWs had children with a risk of vertical transmission. In sub-Saharan Africa, in Togo FSWs had higher HIV prevalence (76.7%) [25]; For unknown reasons, other countries had lower HIV prevalence. It was 32.2% in Kenya, 15.7% in Uganda, 3.5% in Democratic Republic of Congo [25].

The overall prevalence of syphilis of our study was (51.1%) while it was 1.0% in the general population of Rwanda [26]. Although the current prevalence of syphilis in our study could be current or past infection, it is still very high. Due to the high prevalence of HIV in the current study (42.9%), it is not surprising to find a similar prevalence of syphilis. The prevalence of syphilis among FSWs in our study was higher compared to the prevalence of syphilis in many other countries. For instance it was 3.3% in Kisumu, Kenya [10], 21.0% in Kampala, Uganda [3]. However, the prevalence of syphilis among FSWs in Rwanda was similar to the prevalence of syphilis among FSWs in in Addis Ababa, Ethiopia 52.4% [12].

HIV and syphilis have similar modes of transmission. The burden of HIV and syphilis among FSWs is considerable in Rwanda; it could contribute to high morbidity and mortality rates in this key population. The prevalence of syphilis and HIV co-infection was high (27.4%). In terms of proportion of co-infection, in Nepal, the proportion of syphilis infection among HIV-positive FSWs was 31.0% [18], it was 69.2% in our study. In Addis Ababa the proportion of HIV among syphilis-positive FSWs recruited in STIs clinic was 85.1% [12].

Older age was identified as an associated factor of high HIV and syphilis co-infection; the same factor was identified in prevalence of syphilis in a Chinese study [21].

Secondary or higher education was negatively associated with higher prevalence of HIV and syphilis co-infection (aOR = 0.50). The assumption behind this finding may be the fact that FSWs with secondary and higher education could have additional occupation to the sex work, thus reduce the risk of exposure. In addition, they may have possibility to access prevention programmes, and could have power for condom use negotiation. They could also access to STIs treatment compared to illiterate FSWs. In China, researchers also found that years of education was a protective factor of STIs [21].

A recent history of genital sores was identified as an independent variable positively associated with high HIV and syphilis co-infection (aOR = 1.34 [95% CI: 1.05–1.71]). Ulcerative STIs facilitate HIV and other blood borne diseases transmission and acquisition. The same observation regarding HIV infection risk factors was found in India [21].

The prevalence of HBV among FSWs in our study was lower (2.4%). There is no previous estimated prevalence of HBV in the general population or in any other key population including FSWs in Rwanda. HBV prevalence was 8.8% in Brazil [10] whereas in sub-Saharan African countries, for instance in Nigeria, it was 17.1% [17] and it was 13.3% in Nairobi, Kenya [25].

The prevalence of HCV was 1.1% in our study. HCV is not a high burden among FSWs in Rwanda compared to HIV (42.9%) and syphilis (51.1%). Few data on this area are available. However, a study on HCV and HBV conducted among health workers in a tertiary hospital reveled that the prevalence of HBsAg-positive and anti-HCV-positive was respectively 2.9% and 1.3% [27]. Another study conducted among HIV-positive patients in Kigali, Rwanda, showed that HBsAg-positive and anti-HCV-positive was respectively 5.2% and 5.7% [28]. HCV prevalence was similar with the prevalence found in Nairobi, Kenya 0.8% [18], but much lower than the estimated prevalence of HCV in Brazil (23.1%) [10].

Several limitations were encountered in our study. First home-based FSWs and those operating inside hotels were underrepresented in the study because the recruitment was focused on streets and around venues where FSW are found such as hotels, cabarets and night clubs. Second, venues with FSWs less than five per day were excluded by the sampling methodology. Third, specific sexual practices and behaviors and other risk factors associated with STIs among sex workers were not explored in depth. Fourth, the syphilis prevalence reported in this study was first performed using SD Bioline rapid test; confirmed with RPR test. Due to budget constraints a confirmatory test using TPHA test was not performed.

## Conclusion

Based on our findings, we conclude that HIV and syphilis prevalence are very high among FSWs in Rwanda. A strong specific prevention program to avert HIV infection and other STIs transmission to their clients; and proper treatment should be reinforced. Current programs sponsored by the Ministry of Health can help to fight against stigma and discrimination against sex workers – specifically, promoting outreach approach to test and treat all HIV FSWs; testing and treating other STIs among FSWs using rapid tests in their community and improve prevention program for HIV and other STIs reduction. Strengthening HIV and STIs prevention program including condom distribution in the community, training peer educators to be involved in program implementation and linking FSW’s community to care providers in confidential way. Organizing a regular campaign for HIV and STIs testing and treatment in the community. Supporting income generating activities and regular mentorship in order to improve the poor socio-economic status of most of FSWs.

## Abbreviations

Ab: Antibody
Ag: Antigen
AIDS: Acquired Immunodeficiency Syndrome
aOR: Adjusted Odds Ratio
CDC: Centre for disease control and prevention
CI: Confidence Interval
ELISA or EIA: Enzyme-Linked Immuno-Sorbent Assay
FSW: Female sex worker
HBsAg: Hepatitis B surface antigen
HBV: Hepatitis B Virus
HCV: Hepatitis C virus
HIV: Human immunodeficiency virus
IQR: Interquartile range
NRL: National Reference Laboratory
OR: Odds Ratio
PDA: Personal digital assistant
PSU: Primary Sampling Unit
RBC: Rwanda Biomedical Center
RNEC: Rwanda National Ethics Committee
RPR: Rapid plasma reagin
SD: Standard Diagnostic
STI: Sexually Transmitted Infection
TPHA: Treponema pallidum haemaglutination assay
VDT: Venue-day-time
WHO: World Health Organization
